# Supplementary material for: MetaRibo-Seq measures translation in microbiomes
Source: Nat Commun. 2020 Jun 29;11:3268. doi: 10.1038/s41467-020-17081-z (PMC7324362; doi:10.1038/s41467-020-17081-z)
Supplement: Supplementary file 10 — Supplementary Data 7 [file 41467_2020_17081_MOESM10_ESM.zip › File2/Confidence_High_Taxonomy/402887_out.krona.html]

Javascript must be enabled to view this page.

members
magnitude
magnitudeUnassigned
count
unassigned
taxon
rank

402887\_out

44

44
2
superkingdom

976
phylum
44

44
200643
class

44
order
171549

171552
family
43

genus
838
43

40

SRS012989\_contig\_number\_contig-100\_287.66197.66197SRS013638\_contig\_number\_19114SRS014415\_contig\_number\_1770SRS015590\_contig\_number\_13508SRS015794\_contig\_number\_2816SRS017307\_contig\_number\_contig-100\_842.166571SRS019285\_contig\_number\_20SRS019910\_contig\_number\_16316SRS023526\_contig\_number\_12360SRS023715\_contig\_number\_5337SRS043667\_contig\_number\_232SRS043841\_contig\_number\_10801SRS044535\_contig\_number\_12304SRS045713\_contig\_number\_2248SRS046502\_contig\_number\_13659SRS047433\_contig\_number\_53SRS049712\_contig\_number\_16713SRS050752\_contig\_number\_1232SRS053356\_contig\_number\_34144SRS058145\_contig\_number\_contig-100\_250.34853SRS064757\_contig\_number\_21196SRS065397\_contig\_number\_14773SRS077194\_contig\_number\_21649SRS077849\_contig\_number\_12233SRS077991\_contig\_number\_64SRS097958\_contig\_number\_454SRS103987\_contig\_number\_261SRS104036\_contig\_number\_587SRS1041134\_contig\_number\_122SRS1041137\_contig\_number\_25191SRS104327\_contig\_number\_6139SRS105082\_contig\_number\_1819SRS143876\_contig\_number\_23715SRS144297\_contig\_number\_11812SRS144506\_contig\_number\_36702SRS144714\_contig\_number\_23785SRS146764\_contig\_number\_26207SRS147614\_contig\_number\_9946SRS148253\_contig\_number\_6408SRS150029\_contig\_number\_78
species
165179

species
59823

SRS017375\_contig\_number\_7SRS053398\_contig\_number\_13738SRS144603\_contig\_number\_11836
3

1
815
family

816
genus
1

1

SRS012969\_contig\_number\_345
species
821
